# Infectious Etiologies of Childhood Leukemia: Plausibility and Challenges to Proof

**DOI:** 10.1289/ehp.9024

**Published:** 2006-11-30

**Authors:** Siobhán M. O’Connor, Roumiana S. Boneva

**Affiliations:** National Center for Infectious Diseases and National Center for HIV, STD and TB Prevention, Centers for Disease Control and Prevention, Atlanta, Georgia, USA

**Keywords:** ALL, cALL, childhood acute lymphoblastic leukemia, common precursor B-cell ALL, infection, leukemia, virus

## Abstract

Infections as well as environmental exposures are proposed determinants of childhood acute lymphoblastic leukemia (ALL), particularly common precursor B-cell ALL (cALL). Lines of investigation test hypotheses that cALL is a rarer result of common infection, that it results from uncommon infection, or that it ensues from abnormal immune development; perhaps it requires a preceding prenatal or early childhood insult. Ideally, studies should document that particular infections precede leukemia and induce malignant transformation. However, limited detection studies have not directly linked specific human or nonhuman infectious agents with ALL or cALL. Primarily based on surrogate markers of infectious exposure, indirect evidence from ecologic and epidemiologic studies varies widely, but some suggest that infancy or early childhood infectious exposures might protect against childhood ALL or cALL. Several others suggest that maternal infection during pregnancy might increase risk or that certain breast-feeding practices decrease risk. To date, evidence cannot confirm or refute whether at least one infection induces or is a major co-factor for developing ALL or cALL, or perhaps actually protects against disease. Differences in methodology and populations studied may explain some inconsistencies. Other challenges to proof include the likely time lag between infection and diagnosis, the ubiquity of many infections, the influence of age at infection, and the limitations in laboratory assays; small numbers of cases, inaccurate background leukemia rates, and difficulty tracking mobile populations further affect cluster investigations. Nevertheless, existing evidence partially supports plausibility and warrants further investigation into potential infectious determinants of ALL and cALL, particularly in the context of multifactorial or complex systems.

Researchers and physicians have long proposed that infection(s) might cause diverse childhood leukemias. Several lines of scientific evidence support the plausibility of these hypotheses but definitive proof remains elusive. In this overview we highlight the general rationale behind proposals that one or more infections determine or are important co-factors in the development of childhood acute lymphoblastic leukemia (ALL). We briefly outline existing direct and indirect evidence for and against these hypotheses and the challenges to proving infectious causality, even in leukemia clusters. Against a background of assertions that non-microbial environmental exposures and other factors cause ALL, this synopsis draws attention to the very plausible possibility that infection(s), perhaps in the context of multifactorial or complex systems, do contribute to childhood ALL. We underscore the importance of continuing to investigate whether potentially preventable or treatable infections influence the development of childhood ALL, particularly common precursor B-cell ALL (cALL) but also emphasizes the ongoing need for optimal study design and detection tools in this effort.

## Plausibility

Leukemias and lymphomas represent malignant, clonal expansions of white blood cell populations. Simplistically, white blood cells act as the body’s primary defense against infections, where normal, regulated expansions of lymphocytes, monocytes, and neutrophils neutralize and eliminate pathogens. Therefore, aberrant responses to infection(s) could lead to leukemia through the abnormal proliferation of immune cells or a failure to curb the malignant expansion of an abnormal immune cell. Direct infection of lymphocytes might induce unchecked proliferation of a normal or a susceptible (preleukemic) lymphocyte to produce ALL; either persistent infection or malignant transformation triggered by past infection (one cleared from the body some time before ALL is diagnosed) is a conceivable route from direct lymphocyte infection to ALL. Alternatively, infection might indirectly induce ALL by stimulating an overly robust or poorly regulated immune response that would otherwise be protective. Last, other plausible hypotheses suggest that certain patterns of microbial exposure might actually protect against an abnormal lymphocyte proliferation that develops into ALL.

Most childhood leukemias are diagnosed under the age of 8 years, with reports of peak incidences ranging from 2–3 years to 2–5 years [McNally et al. 2003; Ries et al. 1999; Surveillance Epidemiology and End Results (SEER) 2005; U.S. Cancer Statistics Working Group 2005]; cALL accounts for most of these cases. Similarly, many common viral and bacterial infections, including some vaccine preventable infections, occur during early childhood, also exposing susceptible infants, older children, and pregnant mothers (Fleming et al. 1987; Louhiala et al. 1995). Such overlapping age patterns of childhood infection and peak incident cALL suggest that one or more infectious agents might promote childhood cALL. Although observations of higher childhood cALL rates in established economies and higher socioeconomic groups with lower burdens of childhood infection might contradict this idea, a number of immune and demographic factors, such as delayed infection, could reconcile the noted inconsistencies.

In this vein, cALL could represent a relatively rare response to one or more common childhood infections. Only some of many infected children would be at risk for the abnormal immunologic response, acute lymphoblastic leukemia. Age at infection (e.g., very early in life or later than most of the population), preceding or concurrent environmental exposures, and even the child’s cumulative number and types of infections might play critical roles in determining which children ultimately develop cALL. For example, infections early in life might increase or decrease leukemia risk, respectively, by either triggering malignant proliferation or ensuring normal immune development so that later infectious exposures do not produce aberrant lymphoproliferative or immune-mediated syndromes (Bach 2005; Greaves 1988, 1997, 2000).

Alternatively, uncommon or rare agents might induce cALL in many of the small numbers of infected children—existing studies simply may not have detected these microbes or not yet associated them with disease. Current scientific evidence cannot confirm or refute either scenario. For both, it remains unclear whether infection would need to occur after birth, before birth, or if infection during either time period could elicit acute leukemia.

Certain features of microbes reinforce the plausibility of these postulates. Human, animal model, and *in vitro* studies demonstrate that some viruses can incorporate into the human genome, persist in an episomal state, or alter crucial intracellular functions. *In vitro*, Epstein-Barr virus (EBV) transforms B-lymphocytes, creating immortalized cell lines. Intuitively, then, EBV might be capable of inducing B-lymphocyte malignancies such as cALL. However, although a number of studies have associated EBV infection or infectious mononucleosis with solid lymphoproliferative tumors, none have yet linked the virus with leukemia. Irrefutable evidence does confirm that the nonhuman retroviruses avian leukosis virus (ALV), bovine leukemia virus (BLV), feline leukemia virus (FeLV), and murine leukemia virus (MuLV) induce lymphoproliferative disease in birds, cattle, cats, and mice, respectively; all are usually associated with T-cell leukemias. Nevertheless, investigators have never detected FeLV, ALV, BLV, or natural MuLV infection in humans (Klein 2002).

In humans, molecular and immunologic assays can not only detect human T-cell lymphotropic virus type 1 (HTLV-1) in the malignant cells of adult T-cell leukemia/lymphoma (ATLL) but, preceding the development of ATLL, detect the virus in lymphocytes as well as serum antibodies to HTLV-1 (Blattner and Charurat 2005; Centers for Disease Control and Prevention 1993). Epidemiologic studies demonstrate an increased risk for ATLL in HTLV-1 endemic areas, particularly following perinatal infection. *In vitro* studies further reveal that the viral protein Tax can transform or immortalize lymphocytes (Mahieux and Gessain 2003). Yet, ATLL develops in < 5% of individuals infected with HTLV-1 (Blattner and Charurat 2005), and the factor defining which few acquire ATLL remains unclear. If, similarly, cALL occurs in a small percentage of children infected with one or more as yet unconfirmed etiologic microbial agent(s), proving the causal association may be difficult.

Laboratory and epidemiologic evidence also link EBV infection to subsets of Hodgkin disease/Hodgkin lymphoma (HD), a solid tumor malignancy of lymphocytes (Chang et al. 2004a, 2004b, 2005; Glaser et al. 2005; Hjalgrim et al. 2003; Kanzler et al. 2000; Mueller et al. 1989; O’Connor and Scadden 2000). Generally, published molecular analyses have detected the EBV genome in up to 80% of the mixed cellular HD and up to 40–50% of all HD cases examined, but estimates of EBV-infected malignant cells range to > 90%. Although questions on causality remain, some reports have specifically associated HD with a history of EBV-induced infectious mononucleosis, smaller families, or higher socioeconomic status (SES) (Chang et al. 2004a, 2004b; Glaser et al. 2005).

Burkitt lymphoma, however, was the prelude to all confirmed and proposed infectious etiologies of cancers, including childhood leukemias. Epidemiologic data and detection of the virus in tumors consistently implicated EBV as the viral etiology of Burkitt lymphoma in specific regions of sub-Saharan Africa. Although some researchers have recently challenged whether EBV actually induces tumori-genesis, the long-standing association is clear (Johannsen et al. 2005; Niller et al. 2004; Thorley-Lawson and Gross 2004).

Given these examples, researchers have actively pursued the possibility that childhood or maternal/*in utero* infections during pregnancy influence a child’s risk for developing cALL. In 1988, Greaves proposed that reduced exposure to infectious agents during infancy and early childhood might lead to childhood leukemia; essentially, delayed infectious exposure (e.g., lack of child care, small families and/or higher socioeconomic groups) would increase a child’s risk of developing cALL (Gilham et al. 2005; Greaves 1988, 1997, 2000; McNally and Eden 2004). At the same time, Kinlen proposed that a large influx of people into relatively isolated communities (population mixing) might produce mini-epidemics among the previously isolated, immunologically naïve and still susceptible children (Kinlen 1988). The resulting increased exposure of the host population to common or uncommon, potentially leukemogenic infectious agents circulating in the arriving population, might generate increased numbers of leukemia cases over a defined period (Gilham et al. 2005; Kinlen 1988, 1995, 2004; McNally and Eden 2004). While distinct, the two hypotheses would be compatible if an excess of the rarer response, ALL, simply occurs when population mixing exposes a naïve cohort of children to infection at an older age (Gilham et al. 2005). Although initially proposed for situations specific to the United Kingdom, the cornerstones of these hypotheses are relevant to the long-standing, everyday travel and migration patterns inside and across U.S. borders, reduced childhood infections in established economies, increasing global migration, and rising SES in developing or middle-income economies.

Greaves and others also hypothesized that the malignant transformation of a normal cell to leukemia cells requires two separate events, initial priming followed at some point by a secondary incident (Gilham et al. 2005; Greaves 1997, 2000; McNally and Eden 2004; Smith 1997). For example, an *in utero* event (e.g., chromosomal damage induced by maternal environmental exposure or infection; trisomy 21) might produce damaged or preleukemic cells (Canfield et al. 2004; Greaves 1997, 2000; Salomons et al. 1998; Smith 1997; Wiemels et al. 1999a, 1999b, 2001). Later, a postnatal or childhood infectious or environmental exposure, perhaps a common infection, induces leukemia only in those children made susceptible by the primary event.

The preceding hypotheses emphasize that patterns of delayed or unusual childhood infections might interfere with the proper maturation or control of immune cells, producing unchecked, aberrant lymphoproliferation when infection does occur. Interestingly, though, other plausible hypotheses suggest that multiple early infectious exposures might benefit immune development so that later infectious exposures do not trigger aberrant responses, a concept also discussed in the context of immune-mediated syndromes and allergic conditions (Bach 2005). This small body of literature awaits expansion and further testing.

## Direct Evidence for Infectious Etiology

At this time, no reports have identified any specific infectious agent, common or rare, as a cause or co-determinant of childhood ALL or cALL. Only a limited number of published investigations have directly assayed pretreatment specimens for infectious agents at the time of cALL diagnosis and few have retrospectively analyzed archived prediagnostic specimens. More specifically, several small studies failed to detect the human polyomaviruses JC virus and BK virus in leukemia cells or stored newborn (Guthrie) blood spots (MacKenzie et al. 1999; McNally and Eden 2004; Perzova et al. 2000; Priftakis et al. 2003; Smith et al. 1999); one also failed to detect simian virus 40 (Smith et al. 1999). Similarly, a few case–series and case–control studies failed to associate select human herpesviruses or human parvovirus B19 with the diagnosis of ALL (Bogdanovic et al. 2004; Isa et al. 2004; Luppi et al. 1998; MacKenzie et al. 2001; McNally and Eden 2004; O’Connor and Scadden 2000). Most recently, using representational difference analysis one group of investigators detected no specific viral sequences in pretreatment blood or bone marrow leukemic cells from a small number of child and adolescent cALL cases (MacKenzie et al. 2006).

A small number of surveys have also used surrogate measures of animal exposure, such as pet ownership or animal care, to link zoonotic infections with human lymphoproliferative disease (Bross and Gibson 1970; Nowotny et al. 1995; Sordillo et al. 1982). To date, no non-human viruses, retroviruses, or bacteria are associated with naturally occurring human leukemia or lymphoma. It is worth noting, however, that an adverse event of gene therapy raised questions; 2 of 10 children with severe combined immune deficiency (SCID) developed an uncontrolled clonal T-cell proliferation 30 to 34 months after experimental retroviral gene therapy with the MuLV vector (Hacein-Bey-Abina et al. 2003). Interestingly, the T-cell clones of both cases demonstrated vector integration near the hematopoietic protooncogene promoter of the *LMO2* locus, a point insertion rate higher than would be expected with random viral integration. Extensive investigation of these cases, however, detected no replication-competent retrovirus in any cells tested. The investigators concluded that the insertion likely deregulated normal hematopoiesis. It is important that although immune deficiency itself has not been noted as a risk factor for ALL or cALL, medical experiences not infrequently find that immunodeficient or immunocompromised states, including SCID, may predispose individuals to develop lymphoproliferative solid tumors (Biggar et al. 2000).

## Indirect Evidence for Infectious Etiology

Indirect evidence that infections cause or are co-factors in the development of childhood ALL is inconclusive, but much suggests that infectious exposures do influence a child’s risk for ALL or cALL. Briefly outlined here, a number of reviews and reports summarize studies that have attempted to test, prove, or refute infectious determinants of childhood leukemias, particularly cALL (Gilham et al. 2005; Greaves 1997, 2000; Kinlen 1995, 1997, 2004; McNally and Eden 2004; Smith 1997; Smith et al. 1998). Of note, however, very few investigations have directly assayed for active infection, or even past exposure to specific childhood infections (at some time preceding the leukemia diagnosis). Not all have adjusted for confounders of infectious exposure, such as SES.

In general, the results of published epidemiologic and ecologic studies vary, some supportive but others contradictory or inconclusive. Unique characteristics of the diverse populations studied might account for real differences in the findings. Insufficient power, different methodologies, and design weaknesses explain other inconsistencies. Most are retrospective, including the population-based observations and case–control studies. To link infection with childhood acute leukemias, analyses have compared the incidence or prevalence of disease with the following factors: patterns of infection and settings of frequent exposure to infections; patterns of seasonality in the diagnosis of leukemia; time–space clustering of leukemia cases; risk factors for increased susceptibility to childhood infections (e.g., lower immunization rates); factors protecting from infection (e.g., breast-feeding); population density or changes in population density; and differences in SES.

Primarily using surrogate markers for the likelihood of childhood infection (e.g., daycare attendance, number of siblings, contact with father’s workplace, lower SES, less breast-feeding, seasonality) but occasionally analyzing documented clinical syndromes (e.g., ear infection), a number of population-based and smaller epidemiologic studies have now associated delayed or reduced childhood infections with increased risk of childhood leukemia, ALL or cALL (Chan et al. 2002; Gilham et al. 2005; Guise et al. 2005; Infante-Rivard et al. 2000; Jourdan-Da Silva et al. 2004; Ma et al. 2002; McKinney et al. 1999; McNally and Eden 2004; McNally et al. 2003; Perrillat et al. 2002; Petridou et al. 1993; Schüz et al. 1999; Smith et al. 1998; van Steensel-Moll et al. 1986). Yet the specific variables associated with increased risk are often inconsistent, varying between studies; even within single studies, certain markers of infectious exposure are associated with increased leukemia risk, but different markers of similar exposure are not. A few contradictory reports found no increased risk of ALL or cALL with (markers of) reduced or delayed infectious exposures, and some actually described higher risk with more infectious exposures (Chan et al. 2002; Dockerty et al. 1999; Guise et al. 2005; Infante-Rivard et al. 2000; Neglia et al. 2000; Rosenbaum et al. 2000; Schüz et al. 1999). Certain findings also vary—between and within studies—by the type of leukemia and the child’s age at diagnosis. Perhaps consistent with both single- and two-event hypotheses of causation, a limited number of studies and reviews have suggested that maternal infection during pregnancy might increase a child’s risk for developing leukemia (Lehtinen et al. 2003; McKinney 1999 et al; McNally and Eden 2004; Naumburg et al. 2002; Smith 1997; Smith et al. 1999).

Multiple, but not all, ecologic studies have correlated indicators of population mixing and increased population density with increased rates of childhood leukemia (Alexander et al. 1997, 1999; Chan et al. 2002; Gilham et al. 2005; Greaves 1988, 1997, 2000; Hjalmars and Gustafsson 1999; Kinlen 1988, 1995, 1997, 2004; Koushik et al. 2001; McNally and Eden 2004; Ross et al. 1999). Of note, however, retrospective post-hoc analyses have dominated this challenging hypothesis testing, often having to extrapolate from limited population and migration records and assuming unconfirmed patterns of infection transmission and susceptibility. Others have examined seasonal excesses in childhood leukemia or ALL (Badrinath et al. 1997; Dockerty et al. 1999; Fekety and Carey 1969; Gilman et al. 1998; Thorne et al. 1998). Mostly retrospective and with varying results, only some describe seasonal, space–time, or population density clustering and none tested for specific infectious agents (Fekety et al. 1969; Gilman et al. 1999; Gunze and Spears 1968; Gustafsson and Carstensen 1999; Lee 1962).

Other indirect evidence variably suggests that infection(s) might influence the development of childhood ALL. For example, a few investigators have separately examined the effects of vaccination in the U.S. and Finland. The results suggested that immunization against *Haemophilus influenzae* type b, particularly during infancy, might reduce the risk of developing childhood ALL (Groves et al. 1999, 2000). Examining small numbers of childhood ALL cases and non-cases, a few reports have also described potential HLA haplotype differences that might affect susceptibility to infection (Dorak et al. 2002; Taylor et al. 1998).

Overall, widely varying and inconclusive indirect evidence still suggests that infectious exposures do alter a child’s risk for developing ALL. In particular, a level of exposures during infancy and early childhood might protect against childhood ALL. Yet contrasting evidence also suggests that certain types of infections, or infection at certain ages or stages of development, might increase the risk of developing childhood ALL, particularly cALL.

## Unique Challenges to Proof

Studies seeking to prove that one or more infectious agents contribute to the development of childhood ALL or cALL face numerous challenges. Ideally, results should document that a particular infection (or infectious syndrome) precedes the development of leukemia and directly causes (as a primary or secondary factor) the malignant transformation (Knobler et al. 2004). However, only a small portion of the population ever develops childhood ALL. Coupled with limitations in detection assays and the difficulty of prospectively and sequentially screening sufficient numbers of healthy children for preleukemic cells and acute infectious exposures, this characteristic impedes investigations. Therefore, most studies test for infectious agents or past infectious exposures at or after the time of diagnosis. That may be too late to definitively correlate a specific infection with development of ALL, particularly ubiquitous infections that infect many children at a young age. Detecting the responsible agent during diagnosis requires that it persist in the tested tissue(s) and that sensitive and specific detection assays for that agent exist and be correctly employed. Causally linking the agent to leukemia often requires that it be found with greater frequency in cases than in children who do not develop leukemia, unlikely if much of the population is infected during early childhood. On the other hand, detecting an infectious agent in the setting of leukemia does not prove that it caused disease, and failure to detect an agent does not preclude that it induced or promoted the ALL. Posing other investigational challenges, different infectious agents might induce the same abnormal clinical outcome, childhood acute leukemia, through converging pathways of pathogenesis. Confirming causality for each could be difficult. When infection is a critical co-factor (i.e., with environmental exposures or genetics) but not the single cause of leukemogenesis, analyses that focus only on one risk factor may never associate ALL or cALL with infectious etiology.

Childhood leukemia clusters pose additional investigational challenges. If the available background cancer rates are inaccurate, it is impossible to reliably identify an excess of leukemia cases or increased leukemia rates. Clusters are usually small, limiting the power of statistical analyses. Investigations are usually retrospective because the cluster is identified only after someone suspects that an excess number of cases have accumulated over a defined period of time. At that point, most cluster cases have already undergone treatment and many are in remission. Ideally they have no remaining leukemic cells, the cells most likely to harbor a persistent etiologic infectious agent. Indirect markers of past or present infection obtained during the cluster investigation can rarely confirm that infection preceded the diagnosis of leukemia, and retrospective histories inherently have recall bias. Long characteristic of the United States but perhaps increasingly so in other global regions, a highly mobile population with few means to track health care and exposure histories, along with a paucity of large, population-based longitudinal data sets and specimen collections, make testing the population mixing hypothesis difficult, particularly in cluster investigations.

## Conclusions

Collectively, the existing scientific data continue to suggest that infection plays a critical role in the development of childhood ALL and cALL, perhaps in conjunction with other risk factors. However, no specific microbes—viruses, retroviruses, or bacteria—are definitively associated with disease. Nevertheless, enough direct and indirect evidence correlates ALL or cALL with surrogates of infection or even protection from infection to warrant continued investigations.

The key to future investigations may be in complex systems of multifactorial disease (O’Connor et al. 2006), where infection(s) directly or indirectly causes childhood ALL in susceptible individuals—genetically susceptible or made susceptible by causal co-factors such as environmental exposures. The application of improved microbial detection assays, particularly for viral agents, to well-designed studies of sufficient size and timing is needed to reliably link preceding infection with subsequent leukemia. The comprehensive analysis of well-characterized pretreatment specimens from larger numbers of childhood ALL cases and their families (genetically and probably exposure similar) and well-matched controls could begin to correlate ALL with differences in infectious exposures and other risk factors. However, such studies might fail to identify a critical role for past infections (not persistent at the time of diagnosis) in the pathogenesis of ALL. Though costly, large prospective cohort studies that follow children and store serial specimens from birth—perhaps with paired maternal pregnancy specimens—might retrospectively or prospectively be able to correlate past or present, uncommon or common infections with age at exposure, proximity of exposure to ALL diagnosis, and early preleukemic markers. When employing optimal study design and improved detection tools, both types of studies could help elucidate whether preventing or treating certain infections might avoid a portion of childhood ALL, particularly cALL. Alternatively, such studies might uncover whether early infectious exposures are needed to promote a normally functioning immune system and minimize aberrant lymphoproliferative responses such as ALL. Answers to these questions remain important to promoting child health.
